# Nervonic Acid Promotes Skin Regeneration by Enhancing Collagen Synthesis and Suppressing Inflammation

**DOI:** 10.1111/jocd.71241

**Published:** 2026-10-16

**Authors:** Soobeen Chung, Sun Jeong Kim, Sun Jae Kwon, Hong Bae Jeon, Sang Eon Park, Jong Wook Chang

**Affiliations:** ^1^ Cell and Gene Therapy Institute ENCell co. Ltd. Seoul Republic of Korea; ^2^ Cell and Gene Therapy Institute Samsung Medical Center Seoul Republic of Korea; ^3^ Department of Health Sciences and Technology, SAIHST Sungkyunkwan University Seoul Republic of Korea

**Keywords:** anti‐inflammation, collagen synthesis, cosmetics, skin regeneration, wound healing

## Abstract

**Background:**

Skin regeneration is a fundamental biological process that restores tissue integrity and functional homeostasis following tissue injury. Beyond its essential role as a barrier against environmental stressors, effective skin regeneration is critically relevant to aesthetic outcomes and the modulation of cutaneous aging. Nervonic acid (NA) promotes angiogenesis, supports nerve fiber regeneration, and suppresses pro‐inflammatory signaling pathways. However, whether NA enhances dermal fibroblast proliferation, migration, and collagen production, ultimately contributing to skin regeneration, remains unclear.

**Aims:**

This study investigated whether NA promotes proliferation, migration, and collagen production in dermal fibroblasts and modulates inflammatory responses in dermal fibroblasts and macrophages to evaluate its safety and potential as a skin‐regenerative cosmetic ingredient.

**Methods:**

NA safety was evaluated using LDH and CCK‐8 assays, a primary skin irritation test, and the Hen's Egg Test–Chorioallantoic Membrane (HET‐CAM) assay. Regenerative effects were assessed by evaluating wound‐healing activity and collagen production, while anti‐inflammatory effects were examined in lipopolysaccharide (LPS)‐stimulated dermal fibroblasts and macrophages. Phosphorylation of p38 mitogen‐activated protein kinase (MAPK), SMAD2/3, AKT, and mTOR signaling pathways was examined to elucidate the underlying signaling mechanisms.

**Results:**

NA exhibited no cytotoxicity and did not induce irritation in either the primary skin irritation test or HET‐CAM assay. In dermal fibroblasts, NA facilitated wound closure by promoting cell migration. NA increased phosphorylation of p38, SMAD2/3, AKT, and mTOR, while inhibition of AKT signaling attenuated NA‐induced cell migration and proliferation, indicating its involvement in these regenerative effects. NA also increased collagen accumulation by stimulating collagen synthesis and suppressing matrix metalloproteinase 9 (MMP9) expression. Furthermore, NA inhibited pro‐inflammatory cytokine production in LPS‐stimulated dermal fibroblasts and macrophages.

**Conclusion:**

NA promotes regenerative responses in dermal fibroblasts by enhancing cell migration and collagen production, while suppressing pro‐inflammatory cytokine production. It has potential as an active cosmetic ingredient for supporting skin regeneration.

## 1 Introduction

The skin is composed of three major layers: the epidermis, dermis, and hypodermis. The epidermis, which forms the outermost layer of the skin, is composed of approximately 90% keratinocytes, along with melanocytes and several other cell types. The dermis accounts for approximately 90% of the total skin thickness and is composed of fibroblasts and extracellular matrix (ECM) components, such as collagen and elastin, as well as immune cells, nerves, and blood vessels. This layer is known to maintain the overall structure and function of the skin [1, 2].

Skin regeneration is a biological process through which damaged skin repairs injured tissue and restores its original structure and function including inflammatory responses, cell proliferation, and ECM remodeling [3]. Following skin injury, hemostasis is first achieved, after which an inflammatory response is initiated. This inflammatory phase eliminates invading microorganisms [4] and necrotic tissue [5] while providing molecular signals that trigger the subsequent regenerative process [6]. During the regenerative phase, fibroblasts proliferate and migrate into the wound. Fibroblasts, which are activated by cytokines and growth factors, produce extracellular matrix (ECM) components that facilitate tissue reconstruction and regulate the balance between matrix metalloproteinases (MMPs) and their tissue inhibitors, thereby controlling extracellular matrix deposition and remodeling [7]. Concurrently, angiogenesis occurs, leading to the formation of granulation tissue, which provides a vascularized microenvironment essential for effective wound healing and tissue regeneration [8, 9].

Therefore, the regulation of inflammatory processes and ECM synthesis is critical for effective skin regeneration. However, excessive and persistent inflammatory reactions can damage skin tissues and degrade ECM components such as collagen, contributing to skin aging and various skin diseases [10, 11]. Excessive inflammation induces apoptosis of cells at the wound site and leads to abnormal outcomes such as scar formation, destruction of the ECM, and subsequent excessive collagen deposition. In contrast, an appropriately controlled inflammatory response protects the wound from external infection and stimulates the release of cytokines and growth factors. These mediators activate fibroblasts, stimulate collagen synthesis, and facilitate ECM remodeling and angiogenesis, thereby accelerating the wound‐healing process [12, 13, 14].

Nervonic acid (NA) is a long‐chain fatty acid first identified in mammalian nerve tissues and is associated with the maintenance and development of the nervous system [15]. NA promotes angiogenesis, contributing to the protection and regeneration of nerve fibers [16, 17], and has also been reported to inhibit pro‐inflammatory signaling pathways [18].

In a previous study, NA was demonstrated to enhance angiogenesis, improving the survival of transplanted fat tissues, and was also shown to reduce inflammation during fat grafting [19].

However, it remains unclear whether NA promotes the proliferative and migratory activities of resident skin cells and enhances collagen synthesis, ultimately contributing to the regeneration of damaged skin. This study assessed whether NA modulates inflammation and collagen synthesis in dermal fibroblasts, providing a comprehensive evaluation of its safety and potential efficacy as a cosmetic ingredient.

## 2 Materials and Methods

### 2.1 Cell Culture

Human dermal fibroblasts and RAW264.7 murine macrophages were cultured in Dulbecco's Modified Eagle's Medium (DMEM; Biowest, Nuaille, France) containing 10% fetal bovine serum (Gibco, Carlsbad, CA, USA). The culture medium was further supplemented with 100 U/mL penicillin and 100 μg/mL streptomycin (Gibco BRL). Cells were cultured at 37°C under 5% CO_2_ in a humidified incubator.

### 2.2 Lactate Dehydrogenase Assay

Lactate dehydrogenase (LDH) release was quantified using CyQUANT LDH Cytotoxicity Assay Kit (Invitrogen, Thermo Fisher Scientific, Carlsbad, CA, USA). All steps were performed according to the manufacturer's instructions. Overall, 5000 human dermal fibroblasts were seeded in 96‐well plates. NA (Sigma‐Aldrich, St. Louis, MO, USA) was prepared in dimethyl sulfoxide (DMSO, Sigma‐Aldrich) to a final concentration of 50 mM. Cells were treated with NA at the indicated concentrations ranging from 0 to 200 μM for 24 h. The control groups were treated with a volume of DMSO equivalent to that used for NA treatment. Following treatment, a 50 μL aliquot of cell culture supernatant was transferred to a fresh 96‐well plate and mixed in a 1:1 ratio with the LDH reaction mixture. After incubation at 25°C for 30 min in the dark, absorbance was recorded at 490 and 680 nm using a microplate reader (Multiskan SkyHigh; Thermo Fisher Scientific, Waltham, MA, USA). LDH activity was calculated after correcting for background by subtracting the absorbance at 680 nm from that measured at 490 nm. In the LDH assay, the positive lysis control was used to define the maximum LDH release and calculate the normalized LDH release values. However, it was excluded from statistical comparisons among the experimental groups because it represented an artificially induced maximum‐release condition rather than an experimental treatment group.

### 2.3 Cell Counting Kit‐8 Assay

Cell viability and proliferation were quantitatively determined using the Cell Counting Kit‐8 (CCK‐8; Dojindo, Tokyo, Japan) assay in accordance with the manufacturer's guidelines. For experiments evaluating the effect of NA concentration on cell viability, normal human fibroblasts were seeded in 96‐well plates and treated with NA at concentrations of 0, 25, 50, and 100 μM. For experiments evaluating the effect of NA on AKT signaling, normal human fibroblasts were seeded in 96‐well plates and treated with 50 μM NA and/or MK‐2206 at final concentrations of 0.1, 0.5, 1, or 5 μM. Cell viability was assessed at 24 h, whereas cell proliferation was measured at 24, 48, and 72 h post‐treatment. At each time point, cells were incubated with 10 μL of CCK‐8 reagent in 100 μL of serum‐free medium at 37°C for 3 h. Absorbance was measured at 450 nm using a microplate reader (Multiskan SkyHigh; Thermo Fisher Scientific).

### 2.4 Hen's Egg Test‐Chorioallantoic Membrane Assay

The Hen's Egg Test–Chorioallantoic Membrane (HET‐CAM) was performed approximately 50–60 g of fertilized eggs, which were incubated in an automatic incubator at 38.3°C ± 0.2°C and 58% ± 2% humidity for 7 d. On the 8th d of incubation, only fertilized eggs with sufficiently developed CAMs were selected for use. After the eggshell was removed to expose the CAM, 0.9% NaCl (Sigma‐Aldrich) was applied to the eggshell membrane and incubated for up to 30 min to facilitate separation of the eggshell membrane and CAM. Subsequently, the saline was removed, and the eggshell membrane was carefully removed using forceps. NA was dissolved in squalene to a final concentration of 25 μM, and 300 μL of the NA solution was uniformly applied to the exposed CAM surface. Furthermore, 0.9% NaCl was used as the negative control, whereas 0.1 N NaOH and acetone (Sigma‐Aldrich) were used as positive controls. Following application of the test materials, hemorrhage, vascular lysis, and coagulation were recorded at 30 s, 2 min, and 5 min, respectively, to evaluate toxicity. Two fertilized eggs were used for each test material, and the experiment was performed in duplicate.

### 2.5 Skin Primary Irritation Potential Test

The primary irritation test was conducted on 32 Korean men and women aged 19–60 years. After the test site was cleaned with 70% ethanol, NA was applied for 24 h. The first assessment was conducted 20 min after patch removal, while the second assessment was performed 24 h after patch removal. The primary skin stimulation response was evaluated according to the Personal Care Products Council Guidelines [20]. The results of the skin reactions to NA were expressed as the average response and interpreted with reference to previous studies [21].

### 2.6 Wound Healing Assay

Normal human fibroblasts were cultured in six‐well plates at a concentration of 6 × 10 [5] cells/well. The cells were cultured for 48 h until they reached 100% confluence. The center of each well was scratched vertically and rinsed with serum‐free DMEM until the detached cells were completely removed. Subsequently, NA was diluted in serum‐free DMEM to final concentrations of 0, 25, 50, and 100 μM and applied to the cells for 24 h. To investigate the effect of NA on AKT signaling, cells were treated with 50 μM NA and/or 0.5 μM MK‐2206 for 24 h. Wound closure was measured using a microscope (Olympus CKX41; Olympus, Tokyo, Japan) and analyzed using ImageJ (National Institutes of Health, Bethesda, MD, USA).

### 2.7 Real‐Time Quantitative Reverse Transcription PCR

Total RNA was extracted using the AccuPrep Universal RNA Extraction Kit (Bioneer, Daejeon, South Korea), according to the manufacturer's protocol. cDNA was synthesized using ABScript Neo RT Master Mix for qPCR with gDNA Remover (Abclonal, Wuhan, China), following the manufacturer's protocol.

Real‐time quantitative reverse transcription PCR was performed using the QuantStudio 6 Pro Real‐Time PCR System (Thermo Fisher Scientific) and 2× PowerUp SYBR Green Master Mix (Applied Biosystems, Waltham, MA, USA). Each reaction contained 75 ng of cDNA in 10 μL. Thermal cycling was conducted under the following conditions: initial denaturation at 95°C for 10 min, followed by 40 cycles of denaturation at 95°C for 15 s, annealing at 60°C for 30 s, and extension at 72°C for 30 s. Primers were purchased from Bioneer, and their sequences are listed in Tables 1 and 2. Gene expression levels of target genes were normalized to GAPDH. Relative gene expression was then calculated using the 2^−ΔΔCt^ method. All samples were analyzed in triplicate.

**TABLE 1:** Primer sequences for human genes used in quantitative real‐time PCR.

	Forward (5′ → 3′)	Reverse (5′ → 3′)
*COL1A1* [22]	GATTCCCTGGACCTAAAGGTGC	AGCCTCTCCATCTTTGCCAGCA
*MMP1* [23]	ATGAAGCAGCCCAGATGTGGAG	TGGTCCACATCTGCTCTTGGCA
*MMP3* [24]	CACTCACAGACCTGACTCGGTT	AAGCAGGATCACAGTTGGCTGG
*MMP9* [25]	GCCACTACTGTGCCTTTGAGTC	CCCTCAGAGAATCGCCAGTACT
*MKI67* [26]	GAAAGAGTGGCAACCTGCCTTC	GCACCAAGTTTTACTACATCTGCC
*TP53* [27]	CCTCAGCATCTTATCCGAGTGG	TGGATGGTGGTACAGTCAGAGC
*CDKN1A* [27]	AGGTGGACCTGGAGACTCTCAG	TCCTCTTGGAGAAGATCAGCCG
*CDKN2A* [28]	GAAGGTCCCTCAGACATCCCC	CCCTGTAGGACCTTCGGTGAC
*GAPDH* [29]	GAAGGTGAAGGTCGGAGT	TGGCAACAATATCCACTTTACCA
*TNF* [30]	ATGGGCTACAGGCTTGTCACTC	CTCTTCTGCCTGCTGCACTTTG
*IL6* [31]	AGACAGCCACTCACCTCTTCAG	TTCTGCCAGTGCCTCTTTGCTG
*IL8* [32]	GAGAGTGATTGAGAGTGGACCAC	CACAACCCTCTGCACCCAGTTT

**TABLE 2:** Primer sequences for mouse genes used in quantitative real‐time PCR.

	Forward (5′ → 3′)	Reverse (5′ → 3′)
*Tnf*	CCACGCTCTTCTGTCTACT	CAGGCTTGTCACTCGAATTT
*Il6*	CCATCCAGTTGCCTTCTT	CCGACTTGTGAAGTGGTATAG
*Il1b*	CAGGCAGTATCACTCATTGT	AGGATGGGCTCTTCTTCA
*Gapdh* [19]	TTCAACGGCACAGTCAAG	CCAGTAGACTCCACGACATA

### 2.8 Western Blotting

Proteins were isolated using RIPA buffer (BIOSESANG, Seongnam, Korea) containing a protease inhibitor cocktail and ethylenediaminetetraacetic acid (GenDEPOT, Katy, TX, USA). The protein concentration in each sample was determined using the Bradford method (Bio‐Rad, Hercules, CA, USA), and 10 μg of total protein per sample was separated by sodium dodecyl sulfate–polyacrylamide gel electrophoresis. After electrophoretic separation, proteins were immobilized onto polyvinylidene fluoride membranes. The membranes were blocked with 5% skim milk (BD Difco, Franklin Lakes, NJ, USA) in 1× Tris‐buffered saline containing 0.1% Tween 20 (TBST) for 1 h at 25°C. After blocking, the membranes were incubated overnight at 4°C with primary antibodies prepared in TBST. After washing with TBST three times for 10 min each, horseradish peroxidase‐conjugated secondary antibodies were applied to the membranes and incubated for 1 h at 25°C. Protein bands were detected using NICSRO Western Blot ECL substrates (Bionics, Seoul, Korea) and imaged using an iBright CL1500 Imaging System (Thermo Fisher Scientific). The intensity of the protein bands was measured using ImageJ for quantitative analysis.

The following primary antibodies were used: phospho‐p38, p38, phospho‐SMAD2/3, SMAD2/3, AKT, phospho‐mTOR, and mTOR (Cell Signaling Technology, Danvers, MA, USA); phospho‐AKT, phospho‐ERK, and ERK (R&D Systems, Minneapolis, MN, USA); COL1A1 (Novus Biologicals LLC, Centennial, CO, USA); α‐tubulin (Sigma‐Aldrich); and GAPDH (Santa Cruz Biotechnology, Dallas, TX, USA). Horseradish peroxidase‐conjugated anti‐rabbit and anti‐mouse IgG (AbClon, Seoul, Korea) were used as secondary antibodies.

### 2.9 Enzyme‐Linked Immunosorbent Assay

To examine the expression level of COL1A1 following NA treatment, human dermal fibroblasts were plated at a density of 2.4 × 10^4^ cells/well in 12‐well plates. After 48 h of incubation, cells were treated with NA at concentrations of 0, 25, and 50 μM in serum‐free DMEM. Following 24 h of treatment, the culture media were collected and centrifuged at 2000 × g for 10 min. The supernatants were harvested and diluted 1:80 before analysis using the Human Pro‐Collagen I alpha 1 SimpleStep ELISA Kit (Abcam, Cambridge, UK), following the manufacturer's instructions.

To verify the inhibitory effect of NA on inflammation, interleukin (IL)‐6 secretion in the culture media was measured by culturing human dermal fibroblasts in six‐well plates at a density of 1 × 10^5^ cells/well. After 72 h of incubation, cells were treated with 1 μg/mL LPS and NA at concentrations of 0, 25, and 50 μM in serum‐free DMEM. Following 6 h of treatment, culture supernatants were harvested and IL6 levels were analyzed using the Quantikine Human IL6 Immunoassay ELISA Kit, according to the manufacturer's protocol.

### 2.10 Sirius Red Staining

For Sirius red staining, the cells were fixed with 4% paraformaldehyde and rinsed twice with phosphate‐buffered saline. The fixed samples were stained with picrosirius red solution for 1 h in the dark with gentle shaking. After washing twice with distilled water, the samples were rinsed with acetic acid for 1 min to remove unbound dye. Microscopic images were acquired using an Olympus CKX41 microscope (Olympus). The bound dye was extracted by adding 0.1 N NaOH and shaking for 1 h, and its absorbance was measured at 540 nm.

### 2.11 Lipopolysaccharide (LPS)‐Induced Inflammatory Model

Cells were seeded in six‐well plates and cultured until they reached approximately 70%–80% confluence. Human dermal fibroblasts were treated with 1 μg/mL LPS and NA at concentrations of 0, 25, and 50 μM for 2 h. RAW264.7 murine macrophages were pretreated with NA at a concentration of 0, 25, and 50 μM for 2 h, followed by stimulation with 100 ng/mL LPS for 2 h.

### 2.12 Statistical Analysis

Statistical analyses were conducted using GraphPad Prism version 10.2.0 (GraphPad Software, San Diego, CA, USA). Data distribution was assessed for normality using the Shapiro–Wilk test. Differences between two experimental groups were assessed using an unpaired two‐tailed Student's *t*‐test. Differences among multiple groups were assessed using one‐way analysis of variance. Post hoc comparisons were performed using Dunnett's multiple‐comparison tests and Tukey's multiple‐comparison tests. Results are expressed as the mean ± standard deviation, derived from at least three independent biological experiments. Statistical significance was defined as *p* < 0.05 (*), *p* < 0.01 (**), or *p* < 0.001 (***).

## 3 Results

### 3.1 NA Is Non‐Cytotoxic to Dermal Fibroblasts at 25 and 50 μM

To evaluate the cytotoxicity of NA in dermal fibroblasts, LDH and CCK‐8 assays were performed following 24 h of treatment with various concentrations of NA to simultaneously evaluate cell membrane damage and metabolic activity. In dermal fibroblasts, NA at 25 and 50 μM showed no cytotoxicity, whereas NA at 100 and 200 μM resulted in statistically significant but minimal LDH release (4.59% ± 1.72% and 4.30% ± 1.08%, respectively) (Figure 1A). However, CCK‐8 assay results showed that NA did not affect the viability of dermal fibroblasts (Figure 1B). These results indicate that 100 and 200 μM NA exhibit only mild cytotoxicity, which is insufficient to impair overall cell survival or metabolic activity.

**FIGURE 1:**
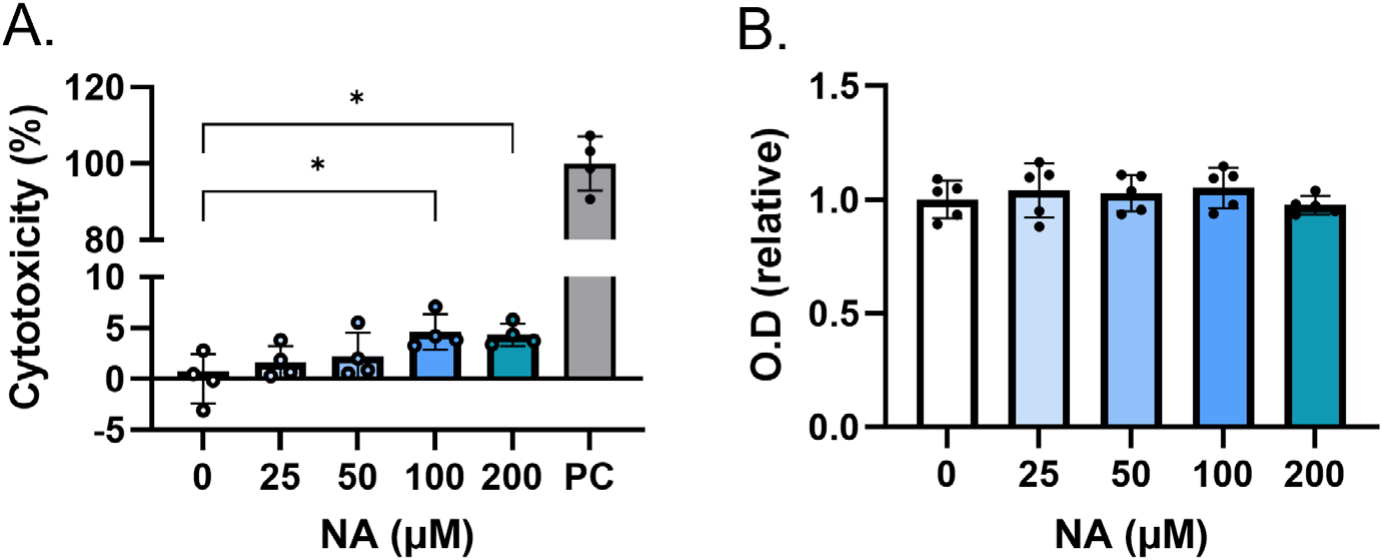
Cytotoxic effects of nervonic acid (NA) on dermal fibroblasts. Cytotoxicity analysis of NA in dermal fibroblast using the (A) LDH assay and (B) CCK‐8 assay. Data are presented as the mean ± SD. Statistical significance was evaluated using one‐way ANOVA, followed by Dunnett's multiple comparisons test to identify specific pairwise differences (*p < 0.05).

Subsequent irritation assays were performed at 25 μM NA. In the HET‐CAM assay, NA at 25 μM did not cause bleeding, vascular lysis, or coagulation, yielding an irritation score (IS) of 0 and being classified as a non‐irritant (Figure 2). Consistent with ex vivo findings, human primary skin irritation potential tests performed with NA at 25 μM revealed no visible erythema or edema in any participants during the observation period (Table 3). Collectively, these findings indicate that NA has a favorable safety profile suitable for cosmetic applications.

**FIGURE 2:**
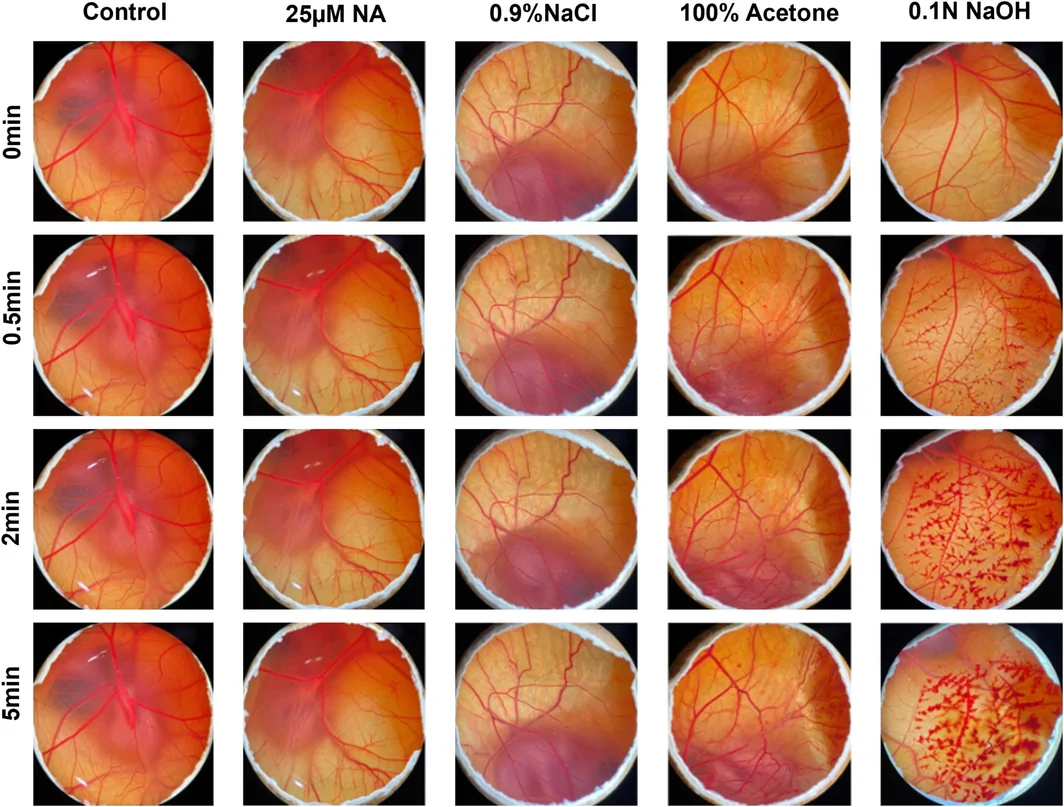
Ex vivo vascular responses observed on the chorioallantoic membrane (CAM). Representative images of the CAM surface were taken before treatment and at 0.5, 2, and 5 min following the application of 25 μM NA (*n* = 2). Saline solution (0.9% NaCl) was used as the negative control, whereas 100% acetone and 0.1 N NaOH were used as positive controls. The negative control group showed no vascular damage, whereas the positive control group showed significant hemorrhage, vascular lysis, and coagulation.

**TABLE 3:** Result of primary skin irritation test (*n* = 32).

Test sample	No. of responder	Assessment								Mean response
		1st				2nd				
		+1	+2	+3	+4	+1	+2	+3	+4	
Nervonic Acid	0	—	—	—	—	—	—	—	—	0.0

### 3.2 NA Promotes Wound Healing in Dermal Fibroblasts

A scratch assay was performed to evaluate the effects of NA on wound healing in dermal fibroblasts. Confluent cells were scratched and treated with NA at 25, 50, and 100 μM to analyze the degree of wound closure after 24 h. Wound closure was significantly enhanced in the 25 and 50 μM NA‐treated groups compared with the control group. However, no significant improvement was observed at 100 μM, and the wound closure rate was similar to or even slightly lower than that of the control (0 μM, 1 ± 0.16; 25 μM, 1.23 ± 0.16; 50 μM, 1.24 ± 0.11; 100 μM, 0.96 ± 0.10; Figure 3A,B). The reduced migration at 100 μM is consistent with the LDH assay results (Figure 1A), suggesting that cellular stress may impair the migration‐promoting effect of NA. These findings indicate that NA promotes wound closure specifically within the non‐cytotoxic concentration range. Therefore, subsequent experiments were focused on the 25 and 50 μM concentrations to exclude the potential effects of cellular stress associated with cytotoxicity at higher concentrations.

**FIGURE 3:**
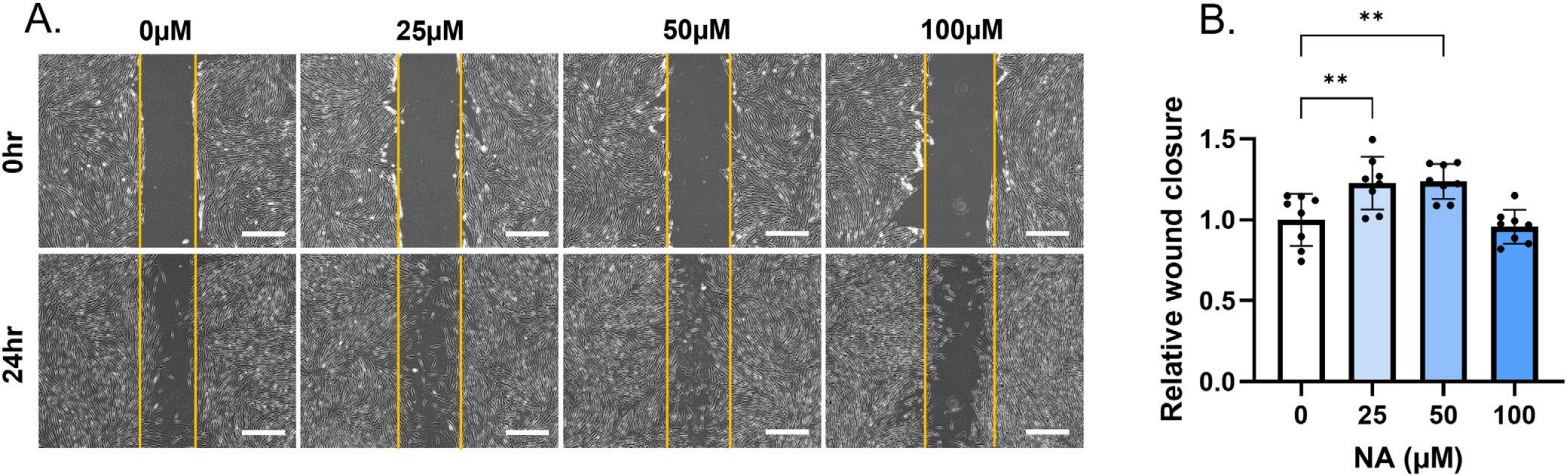
Effect of nervonic acid (NA) on cell migration in dermal fibroblasts. (A) Representative images and (B) quantitative data analysis of wound closure in dermal fibroblasts following NA treatment. Data are presented as the mean ± SD (*n* = 8). Statistical significance was evaluated using one‐way ANOVA, followed by Dunnett's multiple comparisons test to identify specific pairwise differences (**p < 0.01**). Scale bars: 200 μm.

To assess whether the observed NA‐induced increase in wound closure in dermal fibroblasts was due to proliferation or migration, mRNA expression of MKI67, a marker directly associated with the cell cycle, and the mRNA expression of TP53, CDKN1A, and CDKN2A, which are markers of cell cycle arrest and senescence, were analyzed following treatment with NA for 24 and 72 h. No differences in the mRNA expression of MKI67, TP53, CDKN1A, and CDKN2A were observed at 24 h (Figure S1A). The results shown in Figure 1B and Figure S1A suggest that the enhanced wound closure observed at 24 h was induced by cell migration rather than proliferation. However, the expression of MKI67 significantly increased following NA treatment for 72 h (Figure S1B). These results suggest that although NA initially triggers migration, continuous exposure has the potential to promote cell cycle progression in dermal fibroblasts.

### 3.3 NA Contributes to the Regenerative Process via Activation of the p38, SMAD2/3, and AKT/mTOR Pathways

The NA‐induced increase in wound closure in dermal fibroblasts is closely associated with skin regeneration. Therefore, to elucidate the mechanisms whereby NA is involved in skin regeneration during the early and late phases, we examined the phosphorylation of signaling factors involved in collagen synthesis and cell motility. In the early phase, p38 phosphorylation was significantly increased following NA treatment, and SMAD2/3 phosphorylation was elevated in the group treated with 50 μM NA. In contrast, no significant changes were observed in the phosphorylation of ERK, AKT, and mTOR (Figure 4A,B). In the late phase, no significant differences were observed in the phosphorylation of p38 and ERK. In contrast, SMAD2/3, AKT, and mTOR phosphorylation was significantly increased in the group treated with 50 μM NA (Figure 5A,B).

**FIGURE 4:**
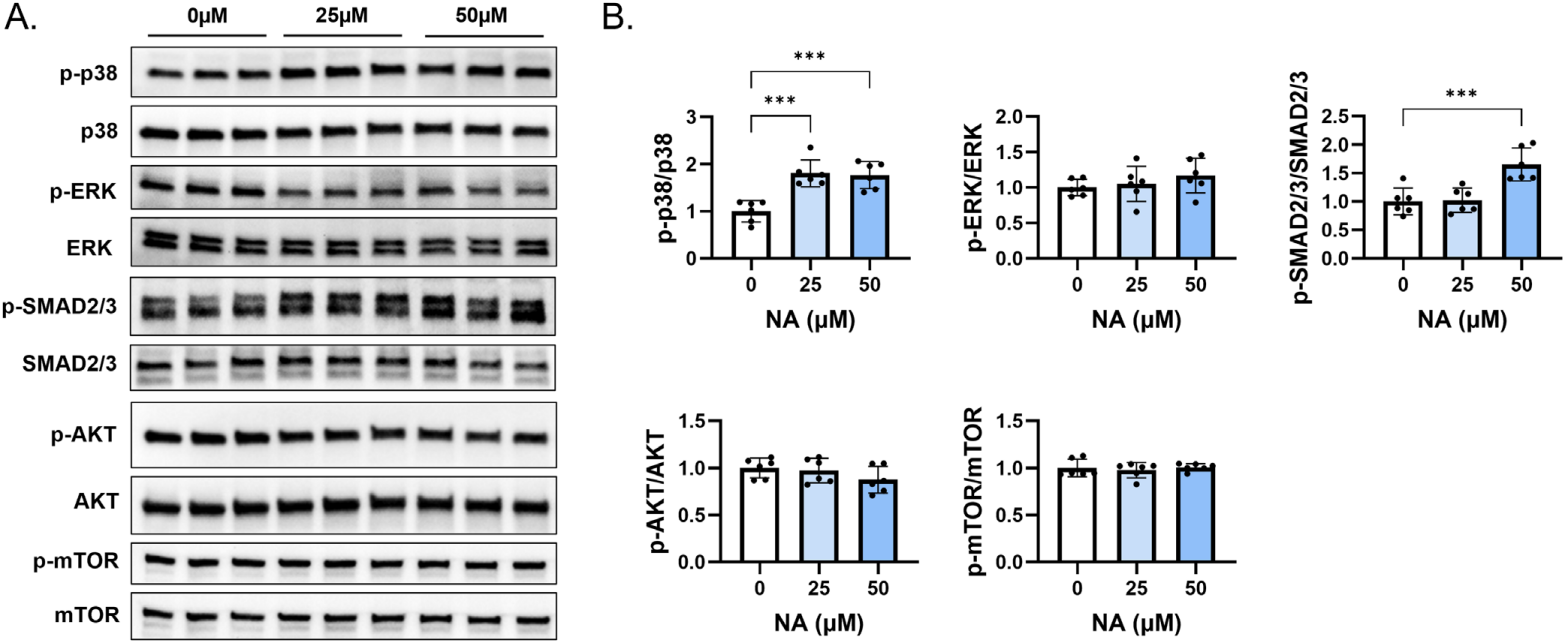
Effects of nervonic acid (NA) on the p38, ERK, SMAD2/3, and AKT–mTOR pathways in dermal fibroblasts during the early phase. (A) Representative Western blot images and (B) quantitative analysis of p‐p38, p38, p‐ERK, ERK, p‐SMAD2/3, SMAD2/3, p‐AKT, AKT, p‐mTOR, and mTOR expression in dermal fibroblasts treated with NA for 0.5 h. Quantified data are presented as the mean ± SD (*n* = 6). Statistical significance was evaluated using one‐way ANOVA, followed by Dunnett's multiple comparisons test to identify specific pairwise differences (***p < 0.001).

**FIGURE 5:**
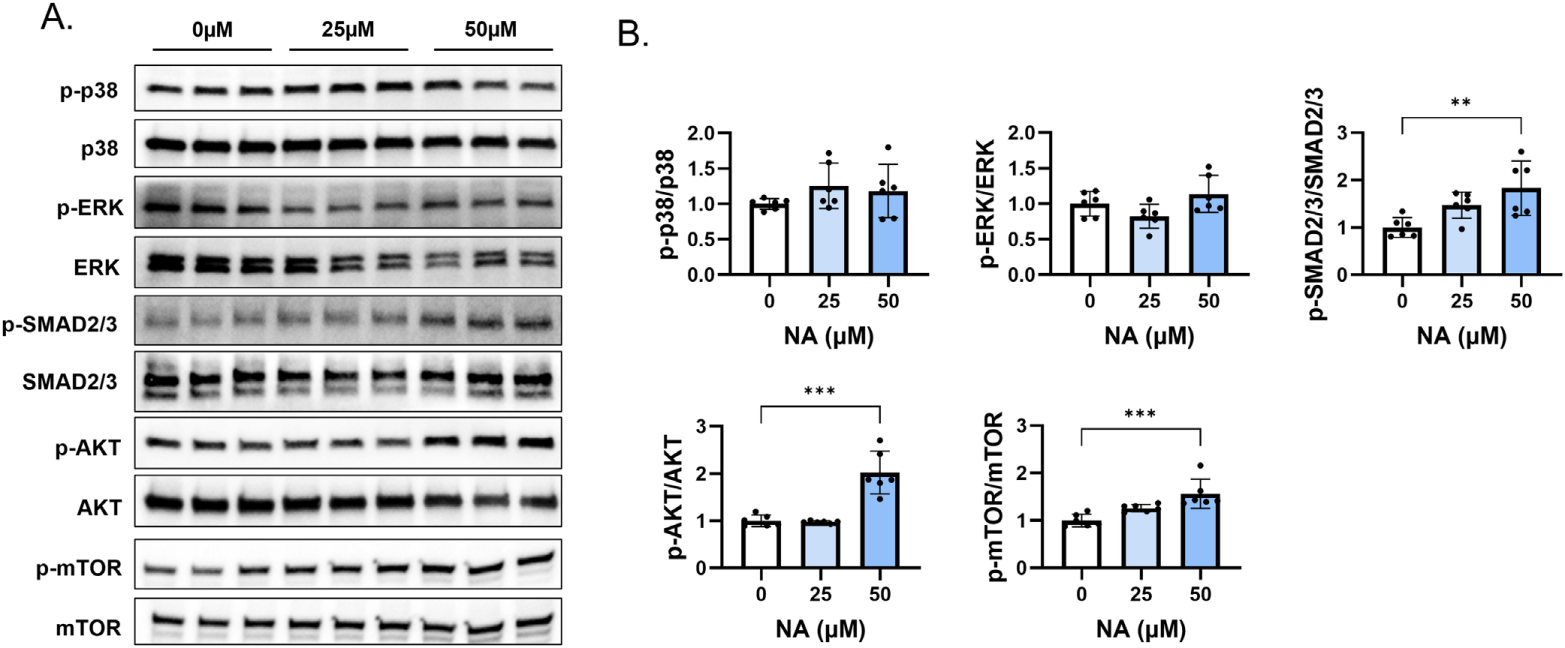
Effects of nervonic acid (NA) on the p38, ERK, SMAD2/3, and AKT–mTOR pathways in dermal fibroblasts during the late phase. (A) Representative Western blot images and (B) quantitative analysis of p‐p38, p38, p‐ERK, ERK, p‐SMAD2/3, SMAD2/3, p‐AKT, AKT, p‐mTOR, and mTOR expression in dermal fibroblasts treated with NA for 24 h. Quantified data are presented as the mean ± SD (*n* = 6). Statistical significance was evaluated using one‐way ANOVA, followed by Dunnett's multiple comparisons test to identify specific pairwise differences (***p < 0.001).

### 3.4 AKT Signaling Functionally Contributes to NA‐Induced Fibroblast Migration and Proliferation

MK2206, an allosteric AKT inhibitor, was used to determine whether AKT signaling functionally contributes to the effects of NA. First, to determine the optimal concentration of MK2206, a CCK‐8 assay was performed to assess cytotoxicity, and AKT phosphorylation was evaluated, confirmed by Western blotting (Figure S2). Based on these results, subsequent experiments were conducted using 0.5 μM MK2206, which inhibited AKT activity without inducing cytotoxicity, and 50 μM NA, which activated AKT signaling. A scratch assay was performed in dermal fibroblasts to evaluate the functional role of AKT in NA‐induced migration. Wound closure was significantly increased in the NA‐treated group compared with the control, MK2206, and MK2206 + NA groups. However, neither the MK2206 group nor the MK2206 + NA group showed a significant difference compared with the control group (Ctrl, 1 ± 0.16; NA, 1.31 ± 0.21; MK2206, 0.94 ± 0.16; MK2206 + NA, 1.08 ± 0.15; Figure 6A,B). The reduction in wound closure caused by AKT inhibition was not rescued by NA treatment, suggesting that NA‐induced migration is mediated by AKT signaling. In the CCK‐8 assay, the effects of NA and MK2206 became more pronounced with increasing treatment duration. NA treatment increased the CCK‐8 signal compared with the control, whereas MK2206 treatment gradually decreased the CCK‐8 signal relative to the control over time. Moreover, the MK2206 + NA group did not differ significantly from the MK2206 group, confirming that NA did not exhibit an additional proliferation‐promoting effect under conditions in which AKT was already suppressed (Figure 6C). These results suggest that AKT signaling is required for NA‐induced fibroblast proliferation.

**FIGURE 6:**
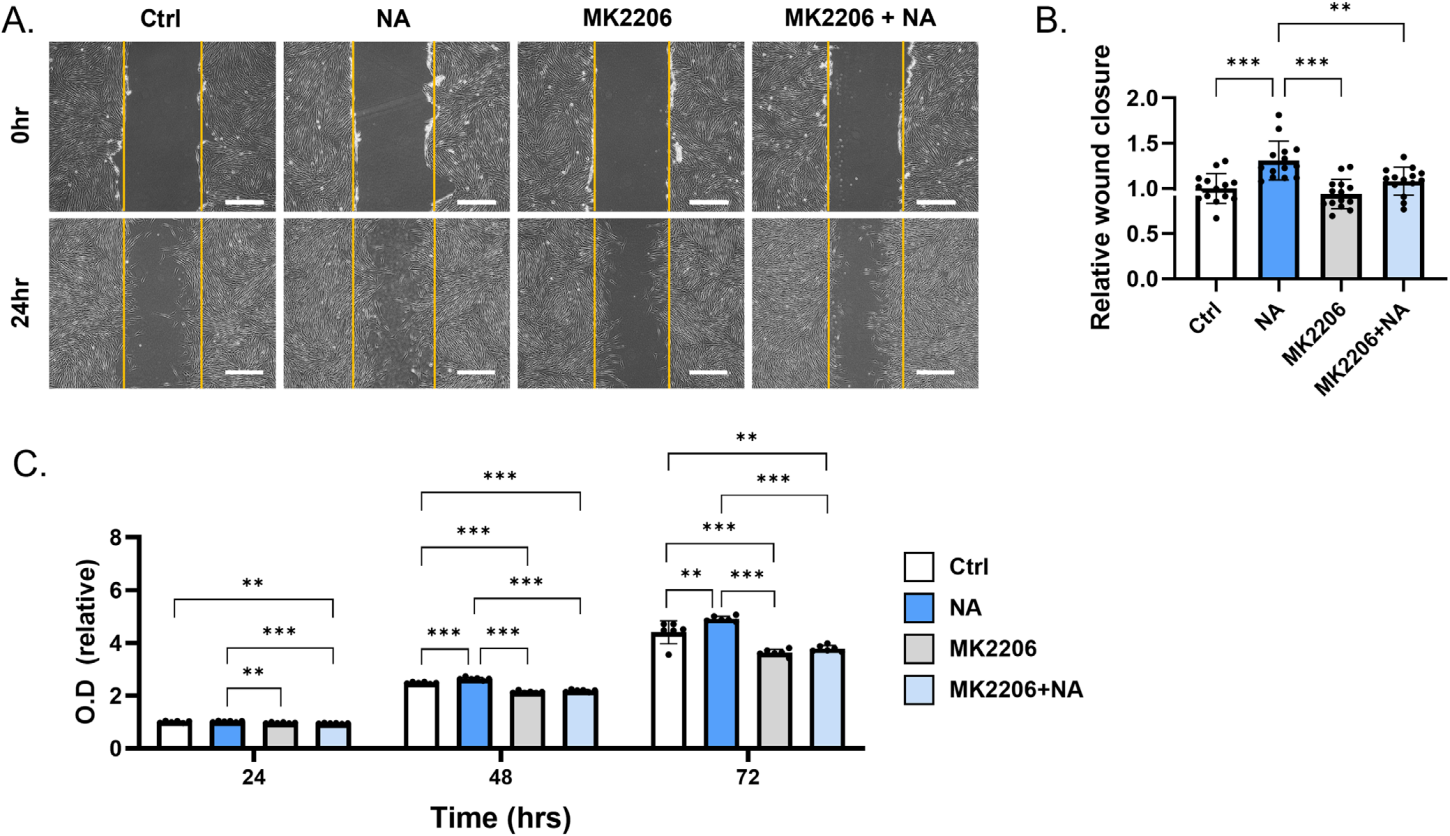
Evaluation of the AKT‐dependent effects of nervonic acid (NA) using MK2206 in dermal fibroblasts. (A) Representative images and (B) quantitative data analysis of wound closure in dermal fibroblasts following treatment with MK2206 and/or NA. Data are presented as the mean ± SD (*n* = 14). (C) Effects of MK2206 and/or NA on dermal fibroblast proliferation, as assessed using the CCK‐8 assay. Data are presented as the mean ± SD (*n* = 6). Statistical significance was evaluated using one‐way ANOVA, followed by Tukey's multiple comparisons test to identify specific pairwise differences (**p < 0.01 and ***p < 0.001). Scale bars: 200 μm.

### 3.5 Collagen Synthesis and Secretion Are Upregulated by NA

To evaluate whether NA treatment was associated with collagen synthesis, a key process in skin regeneration, we analyzed the level of COL1A1 in dermal fibroblasts. The mRNA level of COL1A1 was not affected by NA treatment (Figure 7A); however, the protein level of COL1A1 increased significantly at 50 μM, showing a pattern similar to that of AKT phosphorylation (Figure 7B). This suggests that NA regulates the protein level of COL1A1 through post‐transcriptional mechanisms rather than at the transcriptional level. To confirm whether the increased Type I collagen in cells is secreted extracellularly and contributes to extracellular matrix accumulation, we assessed COL1A1 secretion in the conditioned medium and performed Sirius Red staining on the cells following NA treatment. The results showed that NA treatment induced a modest but significant increase in both COL1A1 secretion (Figure 7C) and extracellular collagen accumulation (Figure 7D). These findings indicate that NA may contribute to regeneration by promoting synthesis and secretion of collagen and increasing extracellular collagen accumulation.

**FIGURE 7:**
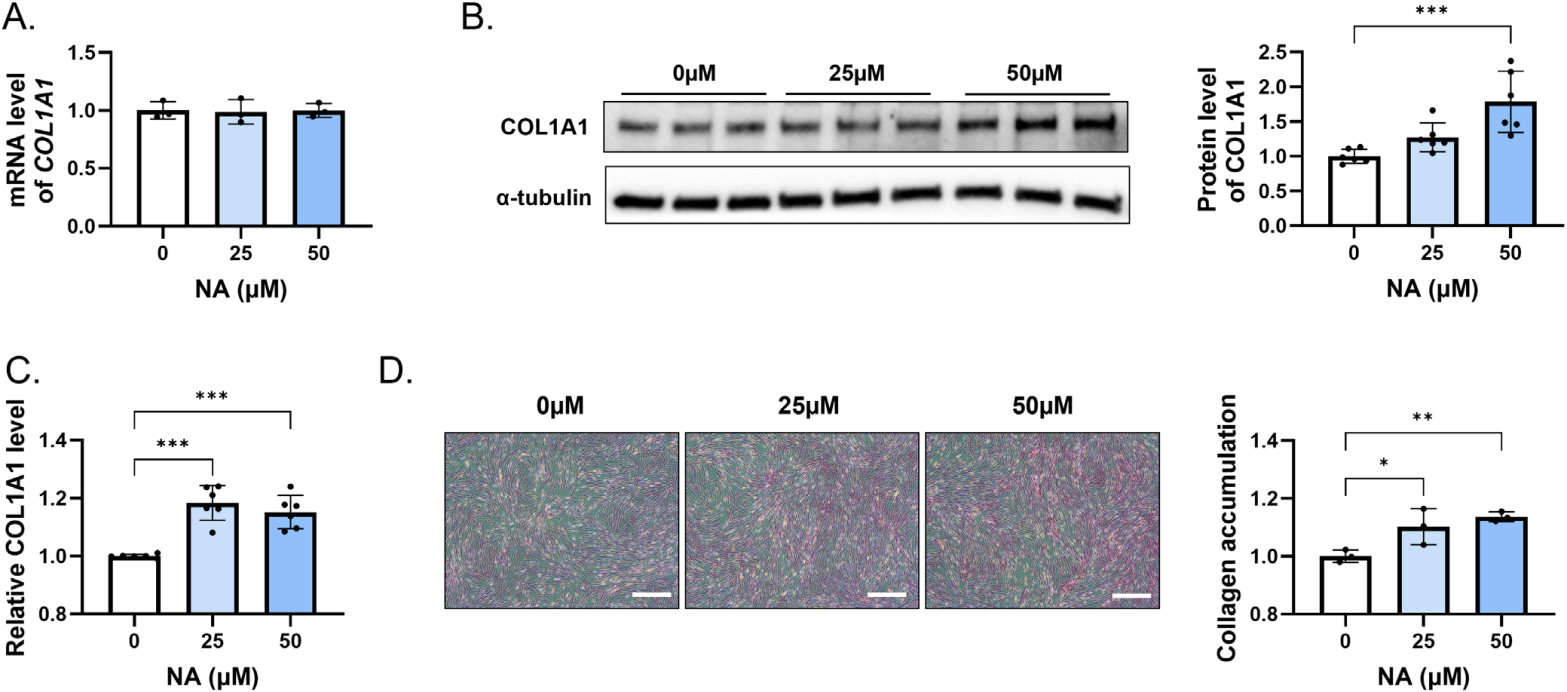
Assessment of collagen synthesis induced by nervonic acid (NA) treatment in dermal fibroblasts. (A) COL1A1 mRNA expression following 6 h of NA treatment. GAPDH expression was used for normalization. Quantified data are expressed as the mean ± SD (*n* = 3). (B) Western blot images and quantitative analysis of COL1A1 expression following 24 h of NA treatment. α‐Tubulin was used as a loading control. Quantitative data are presented as the mean ± SD (*n* = 6). (C) ELISA quantification of COL1A1 secreted into the culture medium by dermal fibroblasts after 24 h of NA treatment. Data are presented as the mean ± SD (*n* = 6). (D) Sirius Red staining and quantification of collagen deposition following 72 h of NA treatment. Quantification of dye retention was performed by measuring absorbance after dye elution. Quantified data represent mean ± SD (*n* = 3). Statistical significance was evaluated using one‐way ANOVA, followed by Dunnett's multiple comparisons test to identify specific pairwise differences (*p < 0.05, **p < 0.01, and ***p < 0.001). Scale bars: 200 μm.

Furthermore, to assess the balance of collagen accumulation, the mRNA expression levels of the collagenases MMP1, MMP3, and MMP9 were evaluated (Figure S3). In dermal fibroblasts, NA did not affect the expression of MMP1 or MMP3 (Figures S3A and S3B), whereas the MMP9 expression was significantly decreased with NA treatment (Figure S3C). This suggests that NA may contribute to collagen accumulation by reducing MMP9‐mediated extracellular matrix degradation. Collectively, NA appears to induce skin regeneration by increasing collagen synthesis and secretion while inhibiting MMP9 expression. These results indicate the potential of NA to promote skin regeneration.

### 3.6 NA Inhibits LPS‐Induced Inflammatory Responses in Dermal Fibroblasts and Macrophages

To evaluate whether NA inhibits inflammation, we established an in vitro inflammatory model by treating dermal fibroblasts and macrophages with LPS. In dermal fibroblasts, the mRNA expression levels of the pro‐inflammatory cytokines TNF, IL6, and IL8, as well as IL6 secretion in the conditioned medium, were measured. LPS treatment increased the mRNA expression levels of TNF, IL6, and IL8, whereas co‐treatment with LPS and NA significantly reduced their expression (Figure 8A). The level of extracellularly secreted IL6 was also enhanced by LPS treatment; however, NA significantly reduced IL6 secretion (Figure 8B). Similarly, in LPS‐stimulated macrophages, NA treatment significantly reduced the mRNA expression levels of Tnf, Il6, and Il1b (Figure 9). These results indicate that NA may exert anti‐inflammatory effects by inhibiting the expression and secretion of inflammatory cytokines in both dermal fibroblasts and macrophages.

**FIGURE 8:**
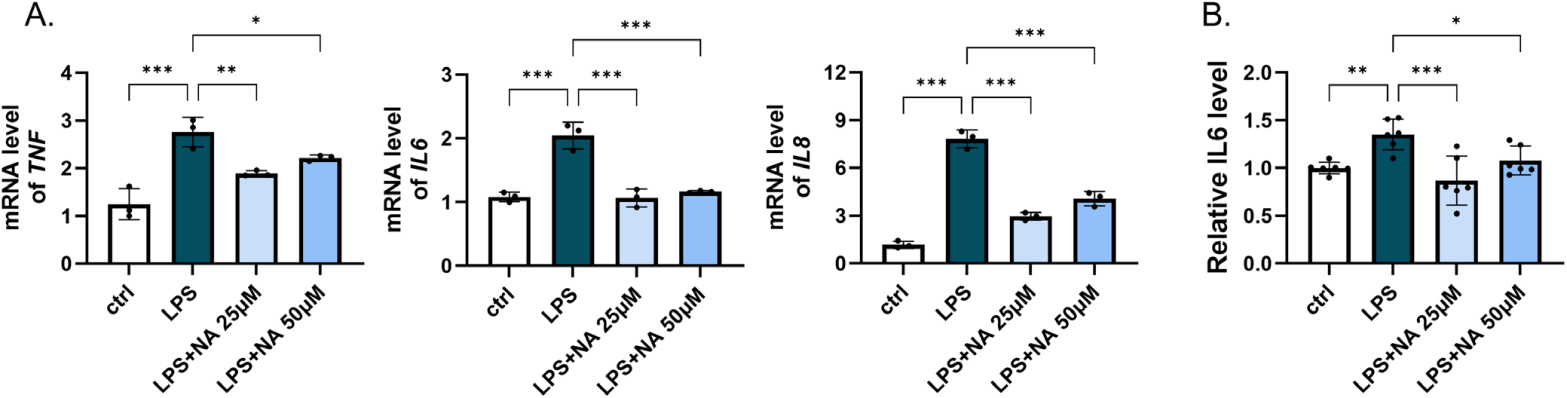
Assessment of the anti‐inflammatory efficacy of nervonic acid (NA) in dermal fibroblasts. (A) qRT‐PCR analysis of the expression of pro‐inflammatory markers (TNF, IL6, IL8) in dermal fibroblasts following 2 h of NA treatment. Data are presented as the mean ± SD (*n* = 3). (B) ELISA quantification of IL6 secreted into the culture medium by dermal fibroblasts after 6 h of NA treatment. Quantified data are presented as the mean ± SD (*n* = 6). Statistical significance was evaluated using one‐way ANOVA, followed by Dunnett's multiple comparisons test to identify specific pairwise differences (*p < 0.05, **p < 0.01, and ***p < 0.001). ELISA, enzyme‐linked immunosorbent assay; qRT‐PCR, real‐time quantitative reverse transcription PCR.

**FIGURE 9:**
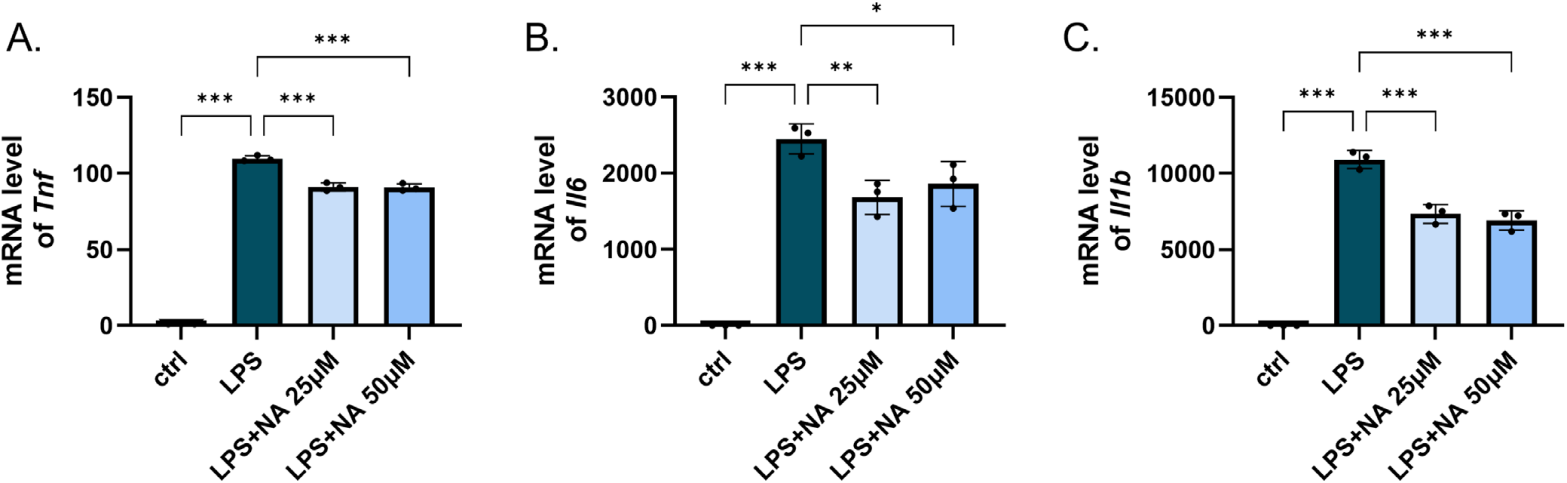
Assessment of the anti‐inflammatory efficacy of nervonic acid (NA) in macrophages. qRT‐PCR analysis to confirm the expression of pro‐inflammatory markers in macrophages following 2 h of NA treatment. mRNA expression of (A) Tnf, (B) Il6, and (C) Il1b. Data are presented as the mean ± SD (*n* = 3). Statistical significance was evaluated using one‐way ANOVA, followed by Dunnett's multiple comparisons test to identify specific pairwise differences (*p < 0.05, **p < 0.01, and ***p < 0.001). qRT‐PCR, real‐time quantitative reverse transcription PCR.

## 4 Discussion

Skin wound healing and tissue regeneration are important targets in cosmetic research, particularly in the context of anti‐aging, scar improvement, and skin barrier recovery [33, 34, 35]. Therefore, various auxiliary substances have emerged to improve skin regeneration and wound healing [36, 37]. This study proposes NA as a novel candidate that may promote wound healing and regulate inflammatory responses without causing cytotoxicity at concentrations of 25 and 50 μM. These effects were observed within a non‐cytotoxic concentration range, and NA did not induce irritation in HET‐CAM assays or human primary stimulation tests, making it suitable for topical applications.

Skin regeneration proceeds through interactions between the epidermis and dermis [38, 39]. When the skin is damaged, keratinocytes rapidly migrate to the injured area and proliferate to cover the wound site and restore the skin barrier [40]. In the dermis, ECM components such as collagen and elastin are synthesized, blood vessels are formed, and growth factors are secreted to structurally stabilize the wounded area and support long‐term regeneration [41]. This study confirmed that NA promotes the migration of dermal fibroblasts and significantly increases collagen protein expression, secretion, and accumulation, while reducing the mRNA expression of MMP9, which is associated with ECM remodeling. These results suggest that NA may promote collagen accumulation by reducing MMP9‐associated ECM degradation. Therefore, NA has the potential to facilitate structural stability and may support accelerated wound recovery by regulating ECM remodeling.

Skin regeneration involves the regulation of cell proliferation, migration, collagen synthesis, and ECM reconstruction and proceeds through the coordinated action of various signaling pathways [39, 42]. Among the MAPK pathways, p38 regulates the inflammatory responses and induces cell migration, collagen synthesis, and ECM remodeling [43, 44, 45], whereas ERK improves wound healing by enhancing migration and proliferation [46, 47]. In addition, SMAD2/3, which TGF‐β activates, contributes to skin regeneration by promoting collagen synthesis and ECM accumulation [48, 49]. The AKT/mTOR pathway contributes to cell proliferation, survival regulation, migration, and ECM protein synthesis during wound healing [50, 51].

Cellular signaling responses to NA treatment exhibited distinct temporal patterns. Phosphorylation of p38 was significantly increased only at the early phase, whereas phosphorylation of AKT and mTOR was increased only at the late phase following treatment with 50 μM NA. No activation of ERK was observed, but SMAD2/3 phosphorylation was increased in the 50 μM NA‐treated group at both early and late phases. These results suggest that p38 may be involved in the early cellular response to NA, while AKT/mTOR signaling may be associated with later cellular responses. In addition, the activation of SMAD2/3 suggests that this signaling may be involved in the ECM‐associated responses induced by NA. However, since cross‐inhibition assays were not performed, it remains unclear whether these signaling pathways are mechanistically linked or independently regulated.

Inhibition of AKT with MK2206 attenuated the NA‐induced increase in wound closure after 24 h, supporting the functional contribution of AKT signaling to fibroblast migration. Since the expression of proliferation‐related markers was unchanged at 24 h of NA treatment and no difference was observed in the CCK‐8 assay, the increase in wound closure observed at this time point appears to be attributable to migration rather than proliferation. On the other hand, MKI67 expression and the CCK‐8 signal were significantly increased after 72 h of NA treatment, indicating the possibility that prolonged NA exposure promotes proliferation in dermal fibroblasts. However, AKT inhibition with MK2206 significantly reduced CCK‐8 signal at 72 h, and NA treatment did not reverse this reduction under conditions in which AKT signaling was suppressed. Taken together, these results suggest that AKT signaling functionally contributes to both NA‐induced fibroblast migration and proliferation.

NA increased intracellular COL1A1 protein expression and secretion without affecting COL1A1 mRNA. These results suggest that NA may regulate the abundance of COL1A1 through post‐transcriptional mechanisms. However, the exact mechanism underlying the NA‐induced increase in COL1A1 protein remains unclear. Therefore, further studies are needed to elucidate this mechanism.

A previous study reported that the addition of NA as an adjuvant during fat transplantation not only induced adipogenesis and angiogenesis but also reduced inflammation, resulting in improved fat transplantation [19]. In this study, NA reduced the mRNA expression of inflammatory markers and IL6 secretion in LPS‐treated dermal fibroblasts. In addition, NA reduced the mRNA expression of LPS‐induced inflammatory markers in macrophages. These observations suggest that NA may possess anti‐inflammatory properties and mitigate the inflammatory response in both dermal fibroblasts and macrophages, potentially fostering a stable regenerative environment.

Taken together, NA showed the potential to promote wound healing, collagen production and accumulation, and inhibit LPS‐induced inflammatory responses under the conditions tested. However, as this study was conducted in vitro, further validation using artificial skin models and subsequent clinical trials is required. Moreover, because the irritation analysis was conducted at a single concentration, further studies using a wider concentration range and finished products are needed to more precisely define the safety margin of NA and evaluate its efficacy throughout practical cosmetic use, including long‐term application stability and formulation feasibility.

In summary, NA promoted wound closure within a non‐cytotoxic range and increased collagen at both the protein and ECM levels in association with the activation of the p38, SMAD2/3, and AKT/mTOR pathways. AKT inhibition experiments further supported that AKT signaling functionally contributes to NA‐induced fibroblast migration and proliferation. Furthermore, in dermal fibroblasts and macrophages, the suppression of NA‐mediated inflammatory marker expression in an LPS‐induced inflammatory environment suggests that NA may help alleviate excessive inflammatory responses caused by skin damage. Additionally, NA did not induce irritation in the HET‐CAM or human primary skin irritation tests, supporting its suitability for safe topical use. These results suggest that NA is a promising cosmetic active ingredient with the potential to promote skin regeneration, increase ECM synthesis, and exert anti‐inflammatory effects. It may potentially be applicable to various functional cosmetic products, including those intended for scar improvement and sensitive skin relief.

## Author Contributions

**Jong Wook Chang:** supervision and writing – review. **Soobeen Chung:** data curation, investigation, visualization and writing – original draft. **Sun Jeong Kim:** conceptualization, formal analysis, investigation, validation and writing – original draft. **Sun Jae Kwon:** writing – review and editing. **Hong Bae Jeon:** funding acquisition and writing – review and editing. **Sang Eon Park:** funding acquisition and writing – review and editing.

## Funding

This work was supported by the National Research Foundation of Korea, 2021R1F1A1064060. Samsung Medical Center, SMX126002.

## Ethics Statement

This study was conducted in accordance with the ethical principles of the Declaration of Helsinki and was reviewed for ethical and scientific validity by the Institutional Review Board (IRB approved number: 1‐220 777‐A‐N‐01‐B‐DICN25264) of DERMAPRO Ltd. The study was carried out in compliance with ethical principles based on the voluntary informed consent of the participants.

## Conflicts of Interest

The authors declare no conflicts of interest.

## Supporting information


**Figure S1:** Effect of nervonic acid (NA) treatment on proliferation and cell cycle arrest in dermal fibroblasts. mRNA expression of MKI67, a proliferation marker, and TP53, CDKN1A, and CDKN2A, which are cell cycle arrest‐related markers at (A) 24 and (B) 72 h after NA treatment. GAPDH was used as an internal control. Quantified data are presented as the mean ± SD (*n* = 3). One‐way ANOVA was conducted to evaluated statistical significance. Dunnett's multiple comparisons test was applied for multiple comparisons (*p < 0.05).
**Figure S2:** Determination of the optimal concentration of the AKT inhibitor MK2206. (A) Cytotoxicity of MK2206 in dermal fibroblasts, as assessed using the CCK‐8 assay. Data presented as the mean ± SD (*n* = 6). Statistical significance was evaluated using one‐way ANOVA, followed by Dunnett's multiple‐comparisons test to identify specific pairwise differences (***p < 0.001). (B) Representative Western blot images of p‐AKT and AKT in dermal fibroblasts treated with MK2206 and/or NA for 0.5 h. (C) Representative Western blot images of p‐AKT and AKT in dermal fibroblasts treated with MK2206 and NA for 24 h.
**Figure S3:** qRT‐PCR analysis of collagenase expression in dermal fibroblasts following 6 h of nervonic acid (NA) treatment. mRNA expression of (A) MMP1, (B) MMP3, and (C) MMP9 at 6 h of NA treatment. GAPDH expression was used for normalization. Quantified data are presented as the mean ± SD (*n* = 3). Statistical significance was evaluated using one‐way ANOVA, followed by Dunnett's multiple‐comparisons test to identify specific pairwise differences (*p < 0.05 and **p < 0.01). qRT‐PCR, real‐time quantitative reverse transcription PCR.

## Data Availability

The datasets used and/or analyzed in the current study are available from the corresponding author upon reasonable request.
