# Supplementary material for: Nervonic Acid Promotes Skin Regeneration by Enhancing Collagen Synthesis and Suppressing Inflammation
Source: J Cosmet Dermatol. 2026 Oct 16;25(10):e71241. doi: 10.1111/jocd.71241 (PMC13673286; doi:10.1111/jocd.71241)
Supplement: Supplementary file 1 [file JOCD-25-e71241-s001.docx]

**Supplementary Figures**

Figure S 1. Effect of nervonic acid (NA) treatment on proliferation and cell cycle arrest in dermal fibroblasts.

Figure S 2. Determination of the optimal concentration of the AKT inhibitor MK2206

**Figure S 3. qRT-PCR analysis of collagenase expression in dermal fibroblasts following 6 h of nervonic acid (NA) treatment**

**Figure Legends**

**Figure S1**. Effect of nervonic acid (NA) treatment on proliferation and cell cycle arrest in dermal fibroblasts

mRNA expression of MKI67, a proliferation marker, and TP53, CDKN1A, and CDKN2A, which are cell cycle arrest-related markers at (A) 24 and (B) 72 h after NA treatment. GAPDH was used as an internal control. Quantified data are presented as the mean ± SD (n = 3). One-way ANOVA was conducted to evaluated statistical significance. Dunnett’s multiple comparisons test was applied for multiple comparisons (*p < 0.05).

**Figure S2**. Determination of the optimal concentration of the AKT inhibitor MK2206

(A) Cytotoxicity of MK2206 in dermal fibroblasts, as assessed using the CCK-8 assay. Data presented as the mean ± SD (n = 6). Statistical significance was evaluated using one-way ANOVA, followed by Dunnett’s multiple-comparisons test to identify specific pairwise differences (***p < 0.001). (B) Representative Western blot images of p-AKT and AKT in dermal fibroblasts treated with MK2206 and/or NA for 0.5 h. (C) Representative Western blot images of p-AKT and AKT in dermal fibroblasts treated with MK2206 and NA for 24 h.

**Figure S3**. qRT-PCR analysis of collagenase expression in dermal fibroblasts following 6 h of nervonic acid (NA) treatment

mRNA expression of (A) MMP1, (B) MMP3, and (C) MMP9 at 6 h of NA treatment. GAPDH expression was used for normalization. Quantified data are presented as the mean ± SD (n = 3). Statistical significance was evaluated using one-way ANOVA, followed by Dunnett’s multiple-comparisons test to identify specific pairwise differences (*p < 0.05 and **p < 0.01). qRT-PCR, real-time quantitative reverse transcription PCR.
